# The immunopathology of B lymphocytes during stroke-induced injury and repair

**DOI:** 10.1007/s00281-022-00971-3

**Published:** 2022-11-29

**Authors:** Mary K. Malone, Thomas A. Ujas, Daimen R. S. Britsch, Katherine M. Cotter, Katie Poinsatte, Ann M. Stowe

**Affiliations:** 1 Department of Neuroscience, The University of Kentucky, Lexington, KY USA; 2 Department of Neurology & Neurotherapeutics, University of Texas Southwestern Medical Center, Dallas, TX USA

**Keywords:** Antibodies, Age-associated B cells, Ischemic stroke, Neuroimmune, Adaptive immunity, Meninges

## Abstract

B cells, also known as B lymphocytes or lymphoid lineage cells, are a historically understudied cell population with regard to brain-related injuries and diseases. However, an increasing number of publications have begun to elucidate the different phenotypes and roles B cells can undertake during central nervous system (CNS) pathology, including following ischemic and hemorrhagic stroke. B cell phenotype is intrinsically linked to function following stroke, as they may be beneficial or detrimental depending on the subset, timing, and microenvironment. Factors such as age, sex, and presence of co-morbidity also influence the behavior of post-stroke B cells. The following review will briefly describe B cells from origination to senescence, explore B cell function by integrating decades of stroke research, differentiate between the known B cell subtypes and their respective activity, discuss some of the physiological influences on B cells as well as the influence of B cells on certain physiological functions, and highlight the differences between B cells in healthy and disease states with particular emphasis in the context of ischemic stroke.

## Introduction

**Fig. 1:**
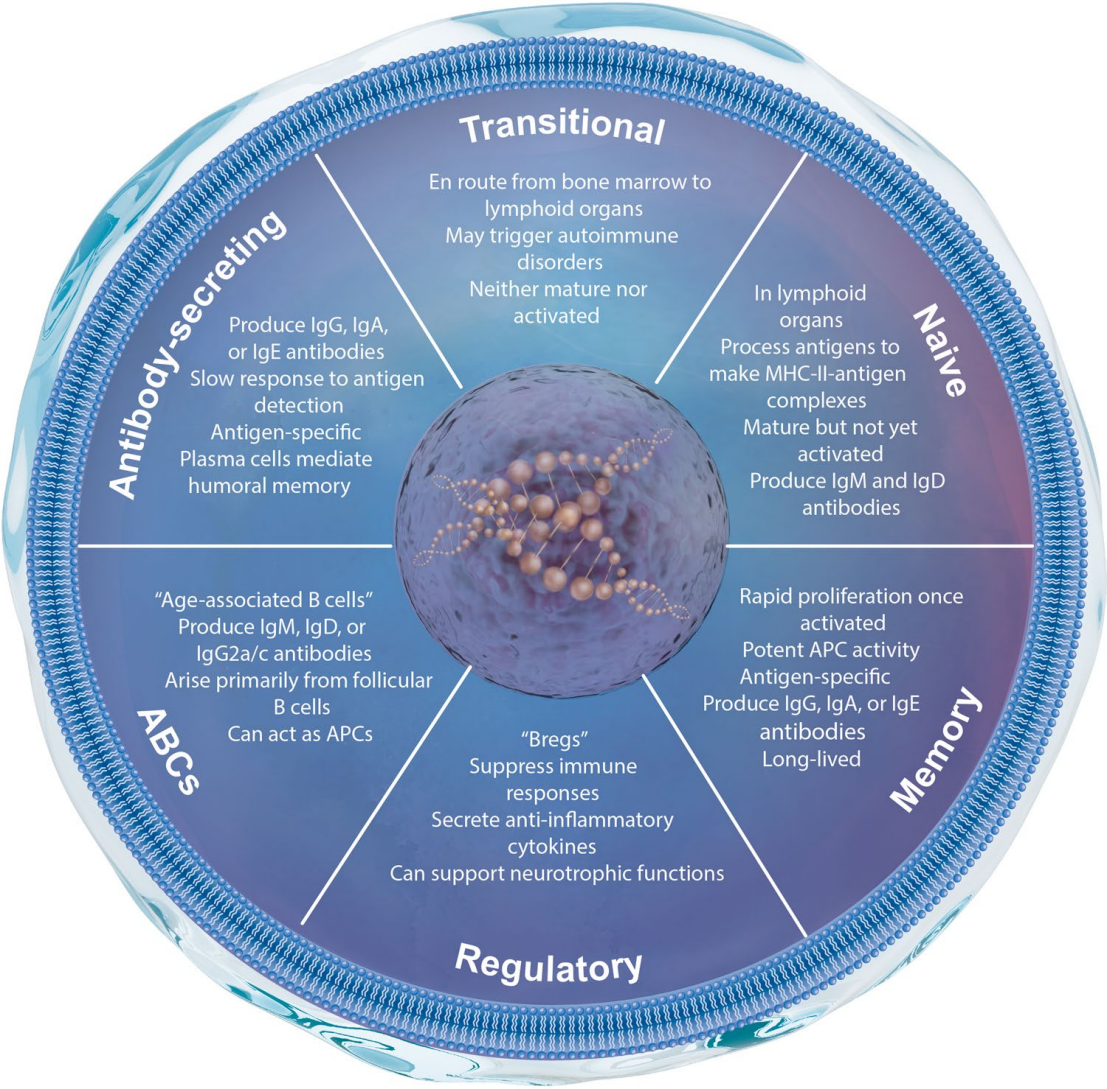
Overview of B cell subsets and their major functions. APCs, antigen-presenting cells

B cells are one of the key players in our adaptive humoral system and can be found throughout the body, from their origin in the bone marrow to secondary lymphoid organs like the spleen, and even the central nervous system (CNS). There are several known subsets of B cells (Fig. 1). B1 B cells develop in the fetal liver and do not require input from the bone marrow, while B2 B cells start from hematopoietic stem cells in the bone marrow and complete maturation after traveling via the peripheral vasculature to secondary lymphoid tissues [86]. These B2 cells can mature into regulatory B cells (Bregs), plasmablasts (antibody(Ab)-secreting), plasma cells, or memory B cells (which are functional during a recurrence of antigen exposure). B cells are called upon or attracted by various pro- and anti-inflammatory chemokines and cytokines, usually to areas of damage or inflammation. It was recently discovered by Brioschi et al. (2021) that there are also meningeal sources of B cells, forming in calvarial bone marrow and traveling to the dura and leptomeninges [6]. It is findings such as these that have continued to disclaim the idea that the CNS is “immune privileged”, though more studies will be needed to determine to what magnitude the CNS pulls B cells from germinal-center-like structures within the meninges. The role of B cells after stroke is complicated, with published studies offering contradicting assertions of whether B cells are beneficial or detrimental to recovery after ischemic injury. The scope of this review will focus on B cells in general health and with co-morbidities relevant to stroke, their role in acute injury and long-term functional recovery after stroke, and how B cells interact in the post-stroke microenvironment predominantly within the CNS.

## An overview of B cells in health and disease

Important aspects of a functioning immune system include response time to infection or injury (ideally rapid and vigorous) and the use of memory cells to optimize future immunity. However, the entire immune system must also be self-limiting. The ability to determine “self” antigens from “non-self” is vital; otherwise, autoimmune reactions and unnecessary inflammation may take place. B cells have roles in many diseases, disorders, and infections, resulting from either B cell functionality regardless of context, or mutations in the B cell receptor (BCR) that affect antigen recognition. In COVID-19 infections, for example, B cell populations see a loss of subtypes, such as transitional B cells, but an increase in double-negative memory B cells, as well as a change in expression of migration markers CXCR3 and CXCR5. Immunoglobulin (Ig)M^+^ or IgG^+^ memory isotypes are generated acutely along with an upregulation of interleukin-6 (IL-6), though these issues resolve in the convalescent period of the disease [31]. More is known about B cells in the context of cancer where they can play contrasting roles. For example, 90% of non-Hodgkin’s lymphomas are B cell-derived [39], and lymphomas can arise at any stage in B cell development, stemming from incorrect rearrangement of the IgH and IgL chains in the BCR [39]. B cells can also be tumor-infiltrating, with (around) two-thirds of infiltrating immune cells consisting of T and B lymphocytes in lung cancers which aid in killing tumor cells [91]. Current studies also examine the potential of B cells as predictors of favorable outcome in cancer, as outlined and reviewed recently by Qin et al. [72], highlighting the complexity of B cell response to disease.

B cells can play a detrimental or supportive role in the aftermath of ischemic injury as well [1, 19, 20, 65], especially depending on co-morbidities such as hypertension, age, and diabetes. Several groups have all reported on the detrimental effects of B cells post-stroke [1, 19, 20, 81], including autoreactivity to myelin basic protein, delayed cognitive deficits, and chronic inflammation [1, 19, 20, 64]. It was also demonstrated that the loss of splenic marginal zone (MZ) B cells contributes to post-stroke infection susceptibility [57], with post-stroke infections often limiting the extent of long-term recovery after stroke. On the other hand, reports found not only that B cells are present in the brain post-stroke, but some subsets support recovery, including IL-10 secreting B cells (i.e., regulatory B cells; Bregs) which provide acute neuroprotection and support neurogenesis [65, 74]. Thus, as with the meninges, our understanding of B cell subsets is still evolving, with a newly identified subset of age-associated CD11b^high^ B cells modulating microglial responses in a transient middle cerebral artery occlusion (tMCAo) model of stroke [46]. Therefore, B lymphocytes cannot be described with a general “post-stroke mechanism,” but instead they become a customized response in the injured CNS that relies on factors including, but not limited to, subset, timing, and location.

### Antibody production

B cell antibody production depends on the cellular subtype, which can be determined by surface receptor expression and signaling threshold. There are 5 antibody isotypes recognized in humans: (Ig)M, IgA, IgG, IgE, and IgD. The IgM monomer and IgD isotypes are capable of functioning as antigen receptors, while the IgM pentamer, IgG, IgA, and IgE are secreted to become circulating antibodies [35]. IgM antibodies are generally part of the initial immune response. In the case of disease states such as stroke, B1 and MZ B cell subtypes can participate in acute and rapid IgM antibody production. IgA antibodies are effective at mounting a response to bacterial infection as well as maintaining homeostasis of gut bacterial colonies [86]. IgE antibodies are typically involved in allergic or anaphylactic response and are usually present at the lowest concentration of the isotypes [35]. The IgD isotype is associated with B cell regulatory processes such as maturation and silencing. Naïve and anergic-phenotype B cells both express similar amounts of IgD on the membrane surface. However, anergic B cells possess a larger reservoir of intracellular IgD and show an increased rate of BCR endocytosis versus naïve B cells, which may explain the reduced responsiveness of anergic B cells [4].

B1 and B2 B cells undergo class switching, altering their antibody production from IgM to IgG, IgA, or IgE. The IgG isotype often comprises most late phases of the initial response and also secondary responses due to class switching, but B cells can also switch to producing polyreactive IgA antibodies. IgA is found in the brain following stroke due to its ability to bind to cellular debris, which is present in the stroke infarct [97]. Some B1 cells can continue to produce natural IgM (IgMn) antibodies that assist in clearance of apoptotic cells [9]. This IgM isotype is polyreactive and binds nonspecifically to T-independent antigens. IgMn can also bind to altered self-antigens, which helps maintain tissue homeostasis and reduce autoimmunity. Understanding the balance of these roles for B cell antibody production is critical, as stroke patients with severe immunosuppression in the acute to subacute phase have a worse post-stroke prognosis [58]. Interestingly, women appear to have higher presence of immunosuppressive factors than men following a stroke [11], highlighting the need for more research into sex differences. Susceptibility in women also increases with age as discussed in the following section.

### The effect of estrogen and sex on B cells in health and stroke

Estrogen plays a vital role in many disease states, including stroke and autoimmunity. In autoimmunity, estrogen, specifically 17 β-estradiol (E2), causes an upregulation of survival factors (e.g., CD22, SHP-1, Bcl-2, and VCAM-1) which allows B cells at the tolerance stage of development to avoid activation of apoptosis because they express estrogen receptors [27]. Therefore, B cells that would normally be destroyed due to recognition of self-antigens are able to survive and proliferate, which leads to an imbalance of immune responses. However, E2 has also been shown to be neuroprotective in several CNS diseases. In experimental autoimmune encephalomyelitis (EAE), a murine model of multiple sclerosis (MS), Bregs that secrete IL-10 modulate the neuroprotection of estrogen pretreatment as this neuroprotection was lost in mice lacking B cells [82]. With regard to stroke, the mortality rate for men versus women at younger ages also shows that pre-menopausal females tend to have better outcomes post-stroke [45], though evidences linking B cells and E2 levels are lacking. While the effect of estrogen on B cells during ischemic events has not been extensively studied, it has been shown that non-nuclear estrogen receptors such as pathway-preferential estrogen receptor-1 (PaPE-1) cause no significant changes in B cell populations during or after ischemic events [83], though further studies need to confirm findings in aged females. This is especially relevant as B cell gene expression is sexually dimorphic, and B cell-derived secretory factors are lost in post-menopausal women [95].

### B cells in aging and immunosenescence

It is well known that as we advance in age, so do our cellular components. Elderly people, especially women [25, 75], have a higher risk for stroke, susceptibility to infections [18], and dementias. This may be due to a chronic, sterile, low-grade inflammation, termed “inflammaging”, which many older individuals are susceptible to developing [25, 29]. The humoral immune system is subjected to this same process, which may result in immunosenescence [55], or the increasing inability for immune cells to respond to infection or other stimuli [21, 96]. While T cells have been studied extensively in aging [59], research into B cells’ role in immunosenescence has remained sparse.

One major hallmark of B cell aging is the loss of B lymphopoiesis within the bone marrow [18, 55]. Normally, the pre-B cell compartment of the bone marrow promotes B cell generation, followed by a series of steps where B cells differentiate, are chosen based on autoreactivity, and then released into the periphery [29, 55]. However, in aging, the pre-BCR and BCR development is affected due to the downregulation or a complete loss of recombination activating genes (RAG) and surrogate light chain (SLC) expression [29, 55], both vital to appropriate B cell maturation. In terms of inflammaging, interferon-γ (IFN-γ), tumor necrosis factor (TNF), and reactive oxygen species (ROS) accumulate in aged mice and humans [66]. Dowery et al. (2021) discovered that the increase of TNF-α, via aging peripheral B cells, plays a mechanistic role in repressing B cell regeneration [18]. TNF-α was also found to stimulate the production of insulin-like growth factor-binding protein-1 (IGFBP-1) which sequesters insulin-like growth factor-1 (IGF-1). This prevents the promotion of B lymphopoiesis within the bone marrow. These studies confirmed a B cell-mediated B cell suppression of lymphopoiesis, as removal of old B cells was followed by a rejuvenation of B cell generation even in aged animals [18].

**Fig. 2:**
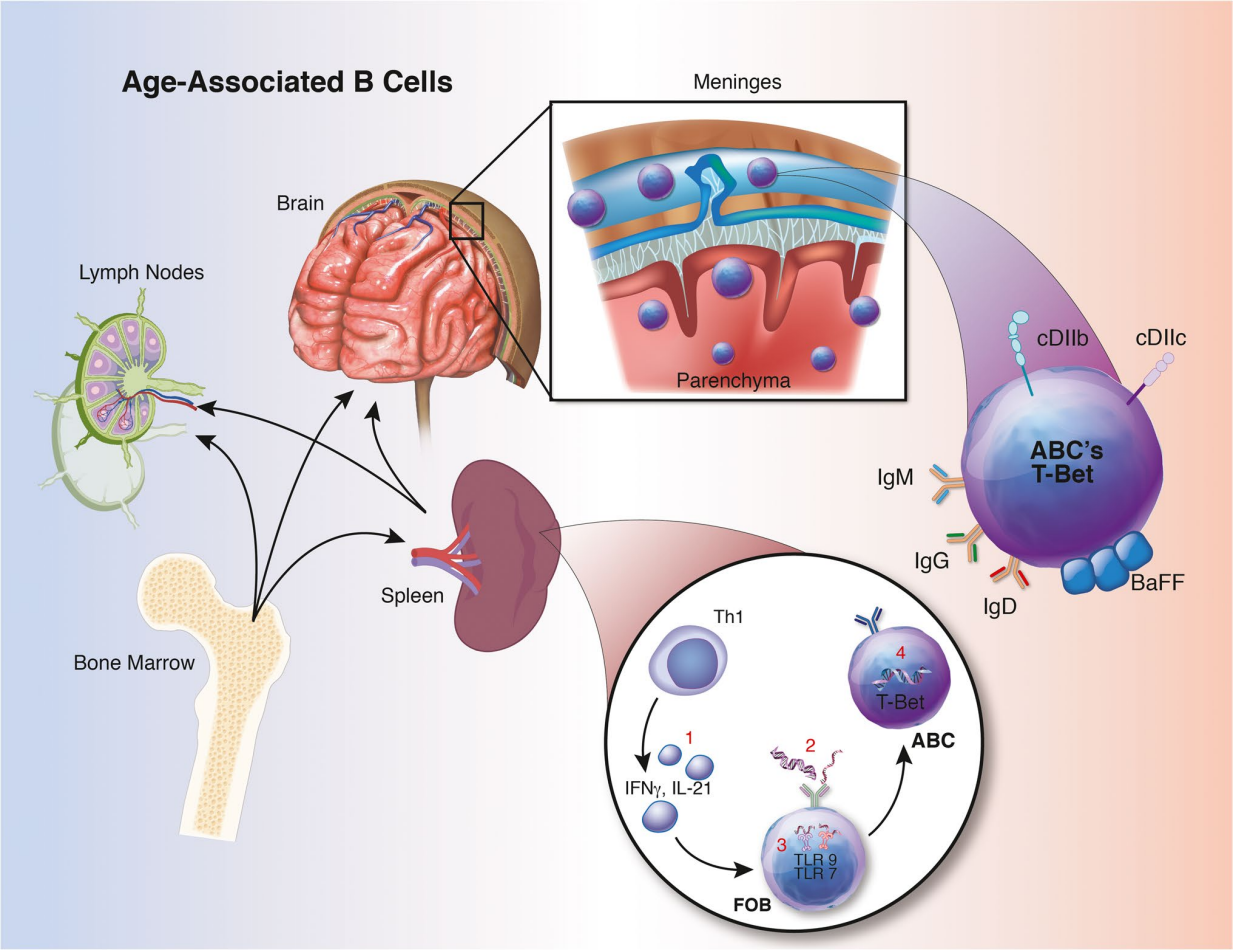
Overview of age-associated B cells (ABCs), including a schematic of development within the spleen: (1) follicular B cells (FOB; also include MZ populations) receive signals from Th1-type cytokines or other nearby interactions, and (2) uptake either endogenous double-stranded (DS)DNA or RNA-associated antigen. (3) These antigens activate toll-like receptor (TLR) 9 and TLR7 and following several divisions and expression of T-bet become an ABC. ABCs can be found in several lymphoid organs, the meninges, and brain parenchyma

A new B cell subset, age-associated B cells (ABCs; Fig. 2), was first described by Hao et al. in 2011 [30]. These B cells form from either follicular or MZ B cells after exhaustive replication but are not created de novo [7, 30, 56]. ABCs respond robustly to stimulation via the BCR and T cell interactions, in combination with toll-like receptor (TLR) 7 or TLR9, IFN-γ, and IL-21 stimulation [7, 30, 56, 100]. However, ABCs act quiescent, meaning that stimulation of this B cell does not promote proliferation. These cells can account for up to 50% of the total mature splenocyte B cell population by 24 months in mice [7]. This subset, which contains several subpopulations, is commonly described as CD19^+^/CD21^−^/CD35^−^/CD23^−^/CD11c^+^/CD11b^high^/T-bet^+^ [7, 30, 44]. ABCs secrete IgG2a/c as well as IgM. IgG2a/c is important in fighting viral infections but can also be autoreactive [7]. ABCs are also known to secrete IL-4, IL-10, IFN-γ, and TNF-α, suggesting both anti- and pro-inflammatory roles, respectively. In stroke and MS, it has been shown that IL-10 reduces neuropathology, resulting in better outcomes for mice and human. Specifically, in stroke, the adoptive transfer of B cells from WT mice reduced infarct volumes compared to IL-10-deficient B cells, confirming additional IL-10-mediated neuroprotection following stroke [65, 74]. In addition to IL-10-mediated neuroprotection in EAE described above, an abundance of IL-10 in human MS patients correlated with lower disability [67]. Some of the first data on how ABCs influence stroke outcomes show that the CD11b^high^ subset can influence microglial phenotypes post-stroke to aid in phagocytosis, although the ABCs themselves can also secrete TNF-α which promotes prolonged neuroinflammation [46]. The balance of immune cells in the periphery and CNS changes drastically over the course of a lifetime, leading to an imbalance of immune responses in areas prone to pathological threats. Overall, the process of aging clearly influences B cell compartments and functions in the population most at-risk for stroke.

## Acute phase of B cell-mediated post-stroke immunopathology:

### Overview of acute injury following stroke onset

The blood–brain barrier (BBB) is located between the brain parenchyma and brain circulation and functions not only to nourish brain tissue, but also filters various compounds from the brain to the blood or cerebrospinal fluid (CSF) for maintaining brain health [38]. Endothelial cells, basement membrane, pericytes, and astrocytes make up the BBB that, along with neurons, form the neurovascular unit. Acute cerebral ischemia produces significant damage to capillary cells, which increases vascular permeability and blood extravasation into the brain parenchyma [85]. The BBB is first disrupted within hours of cerebral ischemia, while the secondary disruption occurs 24–48 h after experimental stroke [43]. The disruption of the BBB is a critical step in the secondary injury cascade and aggravates damage through a variety of mechanisms which allow for peripheral immune cells to enter the brain and augment the neuroinflammatory response more easily. Significant inflammation, leukocyte infiltration, extracellular proteolysis, and vascular activation shortly follow [3]. It is during this 24–48 h post-ischemia phase when the endothelial tight junctions of the BBB, as well as the basement membranes, are destroyed through proteolytic degradation. The BBB’s activated endothelial cells produce adhesion molecules, which attract peripherally circulating immune cells to attach to the endothelium and enter the brain parenchyma. Additionally, danger-associated molecular pattern molecules (DAMPs) and cytokines are released in the brain during the early stages of ischemic damage and enter into systemic circulation via the damaged BBB or through CSF drainage into lymphatic vessels [5], causing inflammation by initiating the immune response in secondary lymphoid organs [22]. Finally, DAMPs proceed to bind to TLRs, scavenger receptors, and receptors on the surface of antigen presenting cells (APC)s for activation of the adaptive immune system [51].

It is important to note that the early proinflammatory response is mostly driven by innate immune cells. In this early acute phase, lymphocytes, such as B and T cells, release cytokines and generate ROS regardless of antigen specificity [41]. In an experimental stroke model, the cytokines IFN-γ, IL-6, and CXCL1 are elevated in the serum within a matter of hours [8], and circulating and splenic immune cells increase the production of IL-6, IL-2, TNF-α, IFN-γ, and CCL2 [63]. Following stroke, it has been shown that matrix metalloproteinases (MMPs) play a main role in altering BBB permeability, especially MMP-2 and MMP-9 [70]. Additionally, these increased MMP-9 levels correlate with the degree of cerebral edema in rodent models of stroke. Clinically, MMP-9 positively correlates with infarct volume and stroke severity [62], as well as worse neurological outcomes post-stroke. Disruption of the BBB is also a pathway for hemorrhagic transformation following ischemia, further exacerbating the magnitude of CNS injury [32].

### Early recruitment of B cells

After a unilateral ischemic stroke in mice, CXCL13, a B cell-recruiting chemokine, is upregulated in cortical vessels of the ischemic hemisphere within 24 h [61, 73]. Cells expressing CXCR5, the receptor for the CXCL13 ligand, have been found in both hemispheres of the brain acutely after unilateral ischemic stroke, suggesting that CXCL13 expression is more widespread rather than localized to the area of infarct. B cells then infiltrate the brain via the vessels bilaterally following stroke [65], and are capable of producing pro- or anti-inflammatory cytokines, depending on subtype, to exert influence on processes including T-cell antigen-dependent inflammation and neuronal survival [98]. Another mechanism by which B cells may infiltrate the CNS is across the blood-cerebrospinal fluid barrier (BCSFB) in the choroid plexus, which occurs in a two-step process [76]. First, B cells must diapedese across capillaries into the choroid plexus stroma and then breach the transepithelial cell barrier to enter the CSF. Unlike the meninges (described below), the migration of B cells across the BCSFB is understudied, and therefore, our understanding of its potential role in stroke is largely speculative. In a healthy adult mouse, very few B cells can be found within the choroid plexus [36]. However, there is evidence that during neuroinflammation, such as in MS, there is an increased number of B cells, particularly plasma cells, in the choroid plexus [60]. Additionally, class-switched memory B cells isolated from MS patients had more pronounced chemotactic activities in an in vitro model, relative to matched healthy controls [28]. In the context of stroke, the choroid plexus has been shown to be a major entry route for another lymphocyte population, T cells, in mice, with choroidal T cells found to increase after stroke [54]. To date, there is no evidence for B cell passage through the choroid plexus into the brain, underscoring the need for further research in this area.

Deletion of B cells via a nonsense mutation in IgM heavy chain (known as the µMT strain) was shown to exacerbate pathology and functional deficits acutely after stroke, without significant alteration to physiological parameters [74]. Specifically, at 1 and 2 days following tMCAo, these B cell-deficient mice exhibited larger infarcts, escalated neurological deficit scores, and increased mortality versus wild-type controls. The ipsilateral hemisphere of the B cell-deficient mice also showed a marked increase of diapedesis of other leukocyte populations. However, a different study in mice lacking B cells (investigating both genetically and pharmacologically-depleted B cells) found contradicting results in similar outcome measures using the same tMCAo paradigm; at 1 and 3 days post-stroke, they observed no difference in edema-corrected infarct volume, nor in number of ipsilateral monocytes and neutrophils in either of the B cell-depleted groups [81]. Again, these studies highlight the complexity of B cell responses after stroke that often leads to contradictory results.

### Neurotrophic capacity of B cells

In addition to an anti-inflammatory function of IL-10 produced by Bregs that diapedese into the post-stroke brain, preclinical studies show that the overexpression of glial, microglial, and endothelial-derived IL-10 reduced infarct volume and apoptosis, while enhancing neurotrophin production within the ischemic hemisphere [15]. Though IL-10 reduces diapedesis and pro-inflammatory chemokine production [68], it may also contribute to neuroprotection by a direct action on cortical neurons. Cortical neurons express IL-10 receptors [24], and in vitro studies showed that IL-10 protects neurons through the activation of multiple pro-survival signal transduction pathways [84], as well as altering neuronal susceptibility to excitotoxicity in a dose-dependent manner [26]. IL-10 cannot cross the BBB, however, so it must be locally produced by the CNS or infiltrating leukocytes, particularly after acute resolution of BBB disruption. It should be noted that IL-10-deficient B cells still display neurotrophic function in an in vitro model of oxygen–glucose deprivation [65], suggesting alternative neurotrophic production. Although leukocytes have the capacity to produce various neurotrophins, B cells are the primary lymphocytic source of brain-derived neurotrophic factor (BDNF), a necessary neurotrophin/neurotrophic factor for neuronal function in both health and disease [90]. In fact, B cells require BDNF for proper development in the bone marrow, BDNF can induce a Ca^2+^ influx into B cells [80], and BDNF protects mature B cells from stress-induced apoptosis [23]. It may be the neurotrophic capacity of B cells that diapedese into the post-stroke brain that induces early protection from ischemic injury in an antigen-independent manner.

### Antibody production during acute injury

After ischemic stroke, the infarct and penumbra become a site of sterile inflammatory response involving a host of cell types. Although the CNS has typically low immune cell populations due to high selectivity of the BBB, ischemic stroke reduces the integrity of the BBB via the breakdown of tight junctions [37]. The innate immune response in the acute phase of stroke initiates recruitment of leukocytes to the brain wherein signals associated with stroke-induced damage activate them. The activation and actions of subsequently recruited lymphocytes are primary drivers of the adaptive immune response through the subacute to chronic phase. After experimental stroke, an adrenergic-mediated loss of splenic MZ B cells contributes to infection susceptibility [57]. Additionally, following cerebral ischemia, unswitched memory B cells were decreased in patients in the acute phase post-stroke but did not correlate with stroke severity. Plasmablasts and other memory B cell populations also remained unchanged in this acute phase post-ischemia cohort. These unswitched memory B cells rapidly mount IgM anti-microbial responses which should be further investigated to see if they drive infection susceptibility in patients [48].

## Chronic phase of B cell-mediated post-stroke immunopathology:

### Chronic establishment of B cells in the post-stroke brain

B cells have been found to remain in the brain as far out as 10 weeks post-stroke in mice [94], and remain elevated peripherally 12 weeks post-stroke in human ischemic stroke patients [50]. It is important to note that few rodent stroke models affect the rodent motor cortex, so most of the models are not ideal for comparing motor function at chronic timepoints that better mimic long-term deficits in those who have had stroke. This is supported by a plethora of publications showing that initial motor deficit differences between experimental and control/wildtype groups tend to disappear within a matter of weeks [12, 53]. Despite robust functional deficit modeling, there remains evidence that B cells could be involved in plasticity and regeneration occurring in motor areas of the brain (in addition to in vitro results described above). Acute (i.e., 4 days post-tMCAo) bilateral diapedesis of B cells in Ortega et al. ([65]) showed elevated B cell migration to five areas associated with motor function in both B cell-depleted (followed by adoptive transfer of B cells) and B cell-sufficient mice, in addition to areas of neurogenesis remote from the tMCAo-induced infarct [65]. Depletion of B cells for weeks after stroke in these studies confirmed that a beneficial effect of B cells on both motor and cognitive recovery was lost with the sustained depletion in young male mice. Outcome measures besides motor function *do* show significant differences when B cell presence/function is manipulated. Doyle et al*.* used a modified MCAo to investigate if B cells played a role in the development of cognitive deficits at chronic timepoints after stroke, and found that both B cell-deficient µMT mice and mice with pharmacological B cell depletion fail to develop the delayed cognitive impairment that WT or control mice displayed at 7 and 12 weeks post stroke [20], as outlined below.

### Effect of B cells on cognition in stroke

Stroke is one of the leading causes of disability worldwide [87], with up to 70% of stroke survivors experiencing deficits in cognitive functioning [17, 87]. Deficits can include impairments in memory, executive functioning, attentional dysfunction, and aphasia depending on the location, number, and type of stroke that occurred [17, 77]. B cell infiltration to the brain in the acute phases after stroke can provide neuroprotection and reduce infarct volume, but can also have a detrimental impact in the chronic phases after stroke, most notably contributing to delayed cognitive impairment [2, 20]. B cell infiltration into the brain can occur in various ways, such as through CXCR5/CXCL13 chemokine interactions with CXCL13 upregulated in cerebrovascular endothelial cells 2–3 days following tMCAo [61]. Upon diapedesis, the B cells subsequently release antibodies into the lesion and surrounding tissue, which results in aggregation within 4–7 weeks post-stroke in mice and leads to delayed cognitive impairment [3]. In this experiment, mice who received an anti-CD20 antibody to deplete circulating B cells did not experience cognitive deficits, signifying a deleterious role successfully countered by immunotherapy [3]. The accelerated cognitive decline in the chronic phase after stroke is present in humans as well [49]*.* As stroke is a major risk factor for the development of vascular dementia, work in Alzheimer’s disease (AD) models confirm that B cells migrate into the brain and secrete IgG to reduce amyloid-β (Aβ) plaques. IgG deposits along the plaques can increase inflammation, thus exacerbating the cognitive impairment experienced [5]. B cell depletion in 3xTgAD mice improved spatial memory in the Morris water maze test [42]. Following stroke, a similar increase of IgG has been seen in the CSF, which indicates increased B cell activation resulting in intrathecal immunoglobulin synthesis [71]. Contrarily, B cell-depleted mice exhibit higher levels of anxiety in open field testing in both injured and non-injured cohorts [7]. What remains to be studied is whether there is a post-stroke population of B cells that remain in the parenchyma, and whether by antibody or cytokine production hasten cognitive decline to the point of dementia.

### Long-term antibody production from ectopic lymphoid structures

Independent experiments have confirmed that infiltrating B cells are not sporadically distributed across the brain after stroke but appear to coalesce in groupings described as ectopic lymphoid structures [20], which have also been observed in other CNS disease states [69]. Local autoantibody production in the ischemic brain was already well established, but more recently, it was shown that the ectopic lymphoid structures are producing mature B cells capable of producing CNS-specific antibodies [2]. Autoantibodies can be immunoreactive to a number of CNS antigens, including MAP2, myelin-derived peptides, NMDA receptors, and other molecules abnormally present due to ischemic damage, resulting neurotoxicity and downstream cell death [14, 37]. Many studies across human patients and experimental models report CNS-specific antigen presence in peripheral lymphoid structures acutely after stroke, likely trafficked in by APC, which contribute to T-cell priming [2, 89]. The potential development of these structures from meningeal B cell pools is described below.

## Other considerations for post-stroke B cell immunopathology

### Changes related to non-ischemic stroke

Much more is known about the ischemic stroke-related immune responses than the immune and inflammatory reactions following hemorrhagic stroke, especially regarding the role of B cells. There is mounting evidence that peripheral immune responses associated with hemorrhagic stroke play an important role in pathophysiology and patient prognosis [78]. However, the details of their immunological interactions in the brain are still poorly understood, indicating an increasing need for more study [78]. In intracerebral hemorrhage (ICH) experimental models, spleen-derived T and B cells exhibited significantly decreased populations regardless of ICH size [34]. Additionally, a study with mixed patient cohorts of ICH and ischemic stroke showed that lower B cell counts, and increased B cell CD86 expression, are linked to worse three-month outcomes [88]. Current observations show that acute lymphopenia and the association with infection risk are linked to poorer outcomes across both ICH and ischemic stroke, as outlined above [88, 93]. The mechanisms behind the increased risk of infections are unclear; however, experimental stroke models show that splenic MZ B cells are innate-like lymphocytes and play a key role in early defense against bacterial infections. When looking at subarachnoid hemorrhage (SAH), acute SAH shows a decrease in natural killer (NK) T cells (NKT), both CD4 and CD8 T cells, and regulatory T cells (Tregs), while showing an increase in natural killer NK cells and B cells in peripheral blood. On days 1, 3, and 6 after the clipping operation for aneurysmal SAH, the B cells found in the poor prognosis patients group showed a sustained decreasing trend. This might show that B cells play a more significant role than other peripheral immune cell subsets in terms of patient prognosis for this subset of hemorrhagic stroke [99].

### Interactions of T cells and B cells in stroke

Interactions between B and CD4 T cells are critical for effective adaptive immunity responses [16, 40]. T and B lymphocytes invade the CNS late in experimental stroke and can be identified for up to 12 weeks, often congregating in clusters in the brain, surrounded by myeloid cells [20]. These ectopic lymphoid structures have been seen in chronic tissue inflammation in autoimmune diseases and facilitate B cell differentiation and antibody production of disease-specific antigens that worsen outcome [13]. Ischemic stroke animal models show that CNS antigens activate both CD4 and CD8 T cells in the cervical lymph nodes and spleen [64]. As a result, the peripheral immune response is altered toward a Th1-like phenotype, which is linked to a worsened neurological prognosis after stroke [1]. B cells can operate as APCs, boosting Th1 and Th17 cell activation and proliferation when the BCR binds its antigen [21]. The antigen, together with the BCR, is internalized and processed into peptide fragments in endosomal and lysosomal vesicles. Accessory enzymes, such as proteases, help in the assembly of these peptide fragments onto major histocompatibility (MHC) class II molecules, which subsequently are trafficked to the surface of the B cell to become an APC. B cells as APCs bring about cognate T and B cell interactions which lead to T cell activation and germinal center reactions, as described above.

Regarding CNS antigens, autoreactive CD4 and CD8 T cells, and CD19 B cells appear as early as 4 days after stroke onset. Mice with large ischemic infarct volumes had early T and B cell autoreactivity to GluN2A, an NMDA receptor subunit, in cells from lymph nodes but not in spleen cells [64]. When looking at lesion volumes in animal models of stroke, combined T and B cell deficiency, as well as selective depletion of CD4 or CD8 T cells, reduce lesion volumes [33, 52]. The use of a myelin oligodendrocyte glycoprotein (MOG) T cell receptor (TCR) transgenic (2D2) mouse model allowed for an investigation of CNS-antigen-specific lymphocyte infiltration post-stroke. Delayed CD4 T cell depletion strongly reduced B cell infiltration at 14 days post-stroke [94]. However, when peripheral CD4 T cell populations were allowed to recover, at days 49 and 72 post-ischemia, both groups had high numbers of B cells in the ischemic hemisphere. The B cell population infiltrated the ischemic hemisphere as soon as the peripheral CD4 T cell population recovered. Thus, demonstrating that B cell infiltration into the CNS is facilitated by CD4 T cell-mediated responses [94].

### B cells in the meninges

While much attention has been given to the diapedesis of leukocytes across the blood–brain barrier, immune cells may also infiltrate the brain by migrating over the blood-meningeal barrier (BMB) and the BCSFB in the choroid plexus [76]. Recent research has greatly advanced our understanding of how these alternative barriers in the meninges and choroid plexus are immunologically vibrant sites of not just B cell migration, but also maturation, tolerance, and dysregulation. The meninges are composed of three layers of connective tissue, the outermost of which is the dura mater. The dura mater contains meningeal blood and lymphatic vessels as well as the dural venous sinuses. In addition to the meninges’ basic structural function as a protective membrane surrounding the CNS, it also functions as a site where immune cells, like B cells, can develop, surveil, and ultimately infiltrate the brain [10]. Meningeal B cells comprise approximately 15–30% of the dural immune cells [6, 47]. The origins of these meningeal B cells are still under investigation, with a population of dural B cells classified as progenitors at the pro-B cell stage that did not influx from the skull bone marrow [79]. In contrast, two publications identified the skull bone marrow as the key origin of dural B cells [6, 92]. Their data support that in young healthy mice, meningeal B cells are derived from local skull bone marrow progenitors that originate in the skull bone calvaria, which contains bone marrow niches for hematopoiesis, then migrate to the meninges through specialized vascular channels running through the calvaria to the base of the sagittal sinus [6]. B cells trafficking within these channels are IgM^−^, indicating an early stage of their development. Once B cells have arrived in the meninges, they predominantly populate the dural sinuses, frequently along the superior sagittal sinus, at the occipital confluence of sinuses, and at the transverse sinus, all sites of abundant lymphatic vessels [79]. B cells in the dural sinuses reside adjacent to IL-17-expressing endothelial cells, while B cells within the sagittal sinuses are also in close contact with *Cxcl12*-expressing fibroblast-like cells, both niches supporting B cell development [92]. Given that early B cells in the dura already express CXCR4, the CXCL12-CXCR4 axis may serve as a homing mechanism by which calvaria-derived B cells are recruited to the meningeal compartment for development.

Further characterization of meningeal B cells with flow cytometry has underscored both the immaturity of these B cells as well as the specificity of this population to the dura [92]. CD19^+^ B cells isolated from the meninges lack membrane expression of IgD and IgM and exhibit limited expression of CD45 and B220. B cells derived from other tissues (spleen, lymph nodes, lungs, and small intestine) did not contain similar immature B cell populations, underscoring that this population is unique to the dura. This population does, however, resemble developing B cells found within the bone marrow, as dural B cells have a similar molecular signature that contains multiple intermediate phenotypes along the developmental continuum of B cells that cannot be found in peripheral B cells. Flow cytometric analysis of dural B cell phenotype and single cell RNA sequencing (scRNA-seq) characterization of this population’s molecular signature was further confirmed by mass cytometry analysis of the dura, blood, and bone marrow, which underscored the developmental heterogeneity of dural B cells [6]. Finally, the development of B cells within the dura mater is evolutionarily conserved; scRNA-seq experiments on B cells from the meninges of non-human primates (*Macaca mulatta)* showed that despite the increased heterogeneity of meningeal B cells in these higher organisms, researchers could reliably annotate clusters of B cells at different developing stages, including pro-B, cycling pre-B, small pre-B, and immature B cells, that could not be found in splenic B cell populations [92].

**Fig. 3:**
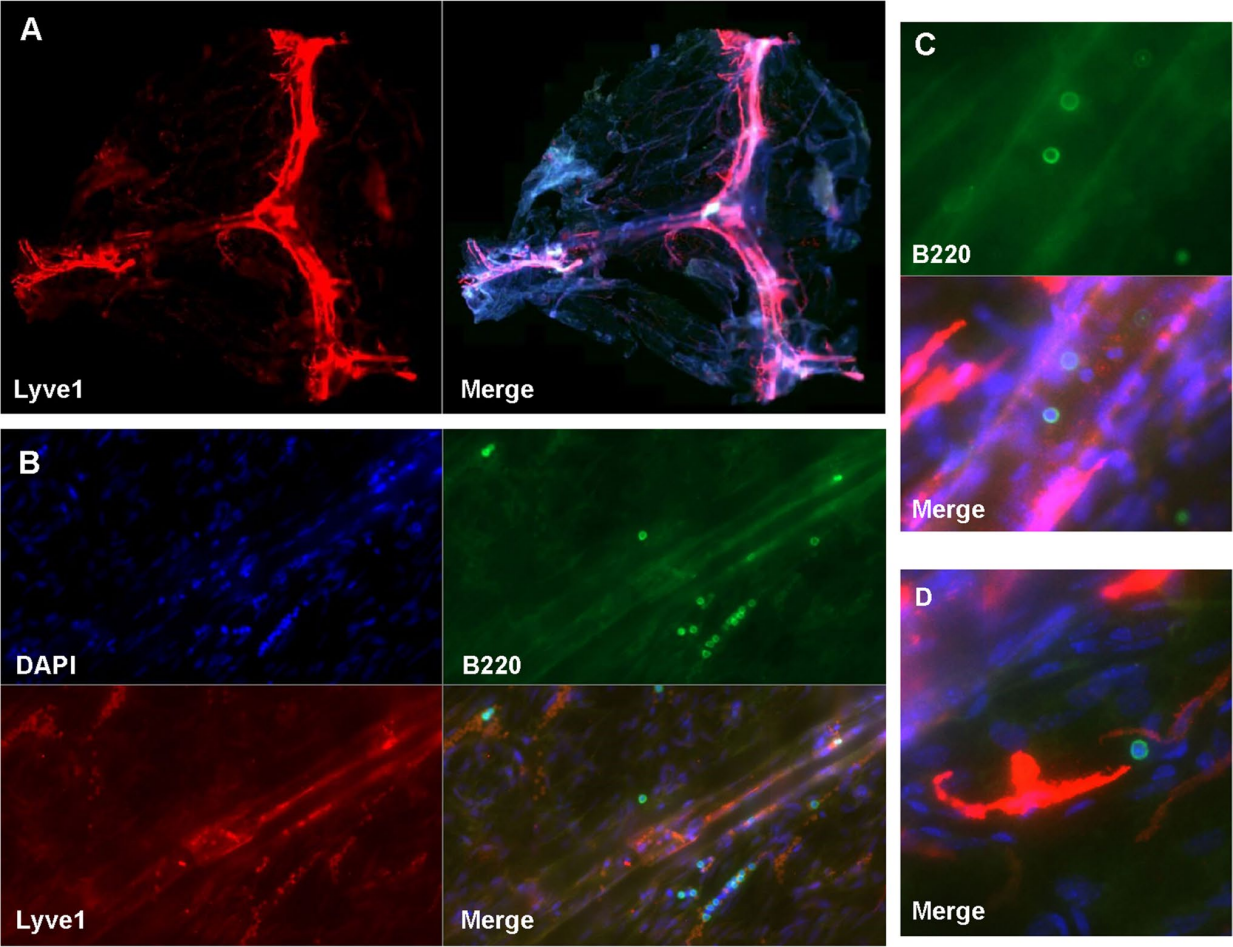
**A** Whole mount of meninges, with lymphatic vessels stained with Lyve1 (red), B cells stained with B220 (green), and nuclei by DAPI (blue) isolated 4 days following a 60-min tMCAo in an 8-week old male C57Bl6/J mouse. **B**–D Magnified images show B cells within lymphatic vessels after stroke. Images acquired on a Axioscan microscope

The origination of meningeal B cells within the calvaria and their immature phenotype within the meninges raises intriguing question about why such localized derivation and development would occur. One possibility is that the meninges, like the bone marrow, serve as a critical location for negative selection, where autoreactive B cell progenitors that recognize self-antigens are eliminated. The meninges as a tolerance checkpoint were specifically tested for CNS antigens using a transgenic mouse line in which 40% of B cells in the bone marrow and spleen are reactive to MOG [92]. MOG-specific B cells were selectively reduced in the meninges relative to the bone marrow of the femur and skull and IgM^+^ meningeal B cells had greater expression of caspase-3, indicating greater apoptosis of MOG-reactive B cells within the meninges. Given these data, it is possible that B cells within the meninges may participate in the development of increased autoreactivity in aging. The total number of B cells markedly increases in the aged dura, particularly ABCs. As mentioned above, ABCs are antigen-experienced, as evidenced by reduced V-region repertoire diversity and an accumulation of somatic mutations. Furthermore, this increased population of ABCs is driven by meningeal infiltration of peripherally derived B cells. Approximately 30% of the dural ABCs were clones shared with the blood. This indicates a switch with aging from the locally derived dural B cells from the calvaria seen in young mice to an age-associated population of dural B cells that trafficked to the meninges from the periphery. The aged dura also exhibits a massive increase in plasma cells along the sagittal sinus, which were predominantly IgM^+^, a change from the smaller, predominantly IgA^+^ population of plasma cells observed in young mice. These age-associated peripheral B cells may differentiate into autoantibody-secreting plasma cells within the meninges, perhaps contributing to the age-related increase in autoreactivity [6] and potentially linked to cognitive decline after stroke. Figure 3 shows a B220^+^ B cell in a Lyve1^+^ sagittal lymphatic vessel at 4 days post-tMCAo in a young male mouse. While not definitive, it does suggest the presence of meningeal B cells after stroke that must be confirmed, and the contribution to pathology investigated, in future studies.

## Conclusion

B cells are key players in many physiological functions, in both healthy individuals and during disease states. With regard to stroke, the contradictory results from the literature indicate that the approaches used to investigate questions related to B cell function (choice of stroke model, method of depletion, functional outcomes, age, sex, and timepoint) hold no small degree of influence on whether B cell activity appears beneficial or detrimental in the post-stroke CNS. Additionally, B cell activity is both influenced by and influential to T cell activity [94]. Based on these facts, we assert that B cell roles are variable and highly dependent on context. A qualification of “good” or “bad” may not be so straightforward or accurate since B cell subtypes can perform very different actions. But while the big picture may still be blurry, the delineation of B cell subsets, specific activity associated with these subtypes, and identification of new tissues of origin and sites of maturation are evolving rapidly to sharpen the perspective on the issue [46, 98].

## References

[1] Becker KJ, Tanzi P, Zierath D (2016). Antibodies to myelin basic protein are associated with cognitive decline after stroke. J Neuroimmunol.

[2] Berchtold D, Weitbrecht L, Meisel C (2019). Friend or foe? – B cells in stroke. Neuroforum.

[3] Bernardo-Castro S, Sousa JA, Bras A (2020). Pathophysiology of blood-brain barrier permeability throughout the different stages of ischemic stroke and its implication on hemorrhagic transformation and recovery. Front Neurol.

[4] Blery M, Tze L, Miosge LA (2006). Essential role of membrane cholesterol in accelerated BCR internalization and uncoupling from NF-kappa B in B cell clonal anergy. J Exp Med.

[5] Bower NI, Hogan BM (2018). Brain drains: new insights into brain clearance pathways from lymphatic biology. J Mol Med (Berl).

[6] Brioschi S, Wang WL, Peng V et al. (2021) Heterogeneity of meningeal B cells reveals a lymphopoietic niche at the CNS borders. Science 373 10.1126/science.abf9277 PMC8448524 34083450

[7] Cancro MP (2020). Age-associated B cells. Annu Rev Immunol.

[8] Chapman KZ, Dale VQ, Denes A (2009). A rapid and transient peripheral inflammatory response precedes brain inflammation after experimental stroke. J Cereb Blood Flow Metab.

[9] Chen Y, Park YB, Patel E (2009). IgM antibodies to apoptosis-associated determinants recruit C1q and enhance dendritic cell phagocytosis of apoptotic cells. J Immunol.

[10] Clatworthy MR (2021). The meninges-a cradle and school for nurturing and educating developing B cells. Immunity.

[11] Conway SE, Roy-O’reilly M, Friedler B (2015). Sex differences and the role of IL-10 in ischemic stroke recovery. Biol Sex Differ.

[12] Corbett D, Carmichael ST, Murphy TH (2017). Enhancing the alignment of the preclinical and clinical stroke recovery research pipeline: consensus-based core recommendations from the stroke recovery and rehabilitation roundtable translational working group. Neurorehabil Neural Repair.

[13] Corsiero E, Nerviani A, Bombardieri M (2016). Ectopic lymphoid structures: powerhouse of autoimmunity. Front Immunol.

[14] Dambinova SA, Khounteev GA, Izykenova GA (2003). Blood test detecting autoantibodies to N-methyl-D-aspartate neuroreceptors for evaluation of patients with transient ischemic attack and stroke. Clin Chem.

[15] De Bilbao F, Arsenijevic D, Moll T (2009). In vivo over-expression of interleukin-10 increases resistance to focal brain ischemia in mice. J Neurochem.

[16] Deenick EK, Chan A, Ma CS (2010). Follicular helper T cell differentiation requires continuous antigen presentation that is independent of unique B cell signaling. Immunity.

[17] Donovan NJ, Kendall DL, Heaton SC (2008). Conceptualizing Functional Cognition in Stroke. Neurorehabil Neural Repair.

[18] Dowery R, Benhamou D, Benchetrit E (2021). Peripheral B cells repress B-cell regeneration in aging through a TNF-α/IGFBP-1/IGF-1 immune-endocrine axis. Blood.

[19] Doyle KP, Buckwalter MS (2017). Does B lymphocyte-mediated autoimmunity contribute to post-stroke dementia?. Brain Behav Immun.

[20] Doyle KP, Quach LN, Sole M (2015). B-lymphocyte-mediated delayed cognitive impairment following stroke. J Neurosci Society for Neuroscience.

[21] Engler-Chiurazzi EB, Monaghan KL, Wan ECK (2020). Role of B cells and the aging brain in stroke recovery and treatment. Geroscience.

[22] Esposito E, Ahn BJ, Shi J (2019). Brain-to-cervical lymph node signaling after stroke. Nat Commun.

[23] Fauchais AL, Lalloue F, Lise MC (2008). Role of endogenous brain-derived neurotrophic factor and sortilin in B cell survival. J Immunol.

[24] Fouda AY, Kozak A, Alhusban A (2013). Anti-inflammatory IL-10 is upregulated in both hemispheres after experimental ischemic stroke: hypertension blunts the response. Exp Transl Stroke Med.

[25] Franceschi C, Garagnani P, Parini P (2018). Inflammaging: a new immune–metabolic viewpoint for age-related diseases. Nat Rev Endocrinol.

[26] Grilli M, Barbieri I, Basudev H (2000). Interleukin-10 modulates neuronal threshold of vulnerability to ischaemic damage. Eur J Neurosci.

[27] Grimaldi CM, Cleary J, Dagtas AS (2002). Estrogen alters thresholds for B cell apoptosis and activation.

[28] Haas J, Rudolph H, Costa L (2020). The choroid plexus is permissive for a preactivated antigen-experienced memory B-cell subset in multiple sclerosis. Front Immunol.

[29] Hagen M, Derudder E (2020). Inflammation and the alteration of B-cell physiology in aging. Gerontology.

[30] Hao Y, O’neill P, Naradikian MS,  (2011). A B-cell subset uniquely responsive to innate stimuli accumulates in aged mice. Blood.

[31] Hartley GE, Edwards ESJ, Aui PM et al. (2020) Rapid generation of durable B cell memory to SARS-CoV-2 spike and nucleocapsid proteins in COVID-19 and convalescence. Sci Immunol 5 10.1126/sciimmunol.abf8891 PMC7877496 33443036

[32] Hong JM, Kim DS, Kim M (2021). Hemorrhagic transformation after ischemic stroke: mechanisms and management. Front Neurol.

[33] Hurn PD, Subramanian S, Parker SM (2007). T- and B-cell-deficient mice with experimental stroke have reduced lesion size and inflammation. J Cereb Blood Flow Metab.

[34] Illanes S, Liesz A, Sun L (2011). Hematoma size as major modulator of the cellular immune system after experimental intracerebral hemorrhage. Neurosci Lett.

[35] J. Gordon Betts KaY, James A. Wise, Eddie Johnson, Brandon Poe, Dean H. Kruse, Oksana Korol, Jody E. Johnson, Mark Womble, Peter Desaix (2013) The adaptive immune response: B-lymphocytes and antibodies. In: Anatomy & Physiology II. OpenStax, Houston, Texas

[36] Jain RW, Yong VW (2021) B cells in central nervous system disease: diversity, locations and pathophysiology. Nat Rev Immunol 10.1038/s41577-021-00652-6 PMC8667979 34903877

[37] Javidi E, Magnus T (2019). Autoimmunity after ischemic stroke and brain injury. Front Immunol.

[38] Kadry H, Noorani B, Cucullo L (2020). A blood-brain barrier overview on structure, function, impairment, and biomarkers of integrity. Fluids Barriers CNS.

[39] Ke J, Chelvarajan RL, Sindhava V (2009). Anomalous constitutive Src kinase activity promotes B lymphoma survival and growth. Mol Cancer.

[40] Kerfoot M, Steven YG, Patel R, Jaymin,  (2011). Germinal center B cell and T follicular helper cell development initiates in the interfollicular zone. Immunity.

[41] Kim JY, Kawabori M, Yenari MA (2014). Innate inflammatory responses in stroke: mechanisms and potential therapeutic targets. Curr Med Chem.

[42] Kim K, Wang X, Ragonnaud E et al. (2021) Therapeutic B-cell depletion reverses progression of Alzheimer’s disease. Nature Communications 12 10.1038/s41467-021-22479-4 PMC8042032 33846335

[43] Knowland D, Arac A, Sekiguchi KJ (2014). Stepwise recruitment of transcellular and paracellular pathways underlies blood-brain barrier breakdown in stroke. Neuron.

[44] Knox JJ, Myles A, Cancro MP (2019). T-bet+memory B cells: generation, function, and fate. Immunol Rev.

[45] Koellhoffer EC, Mccullough LD (2013). The effects of estrogen in ischemic stroke. Transl Stroke Res.

[46] Korf JM, Honarpisheh P, Mohan EC et al. (2022) CD11b(high) B cells increase after stroke and regulate microglia. J Immunol 10.4049/jimmunol.2100884 PMC9446461 35732342

[47] Korin B, Ben-Shaanan TL, Schiller M (2017). High-dimensional, single-cell characterization of the brain’s immune compartment. Nat Neurosci.

[48] Krishnan S, O’boyle C, Smith CJ,  (2021). A hyperacute immune map of ischaemic stroke patients reveals alterations to circulating innate and adaptive cells. Clin Exp Immunol.

[49] Levine DA, Galecki AT, Langa KM (2015). Trajectory of cognitive decline after incident stroke. JAMA.

[50] Li S, Huang Y, Liu Y (2021). Change and predictive ability of circulating immunoregulatory lymphocytes in long-term outcomes of acute ischemic stroke. J Cereb Blood Flow Metab.

[51] Liesz A, Dalpke A, Mracsko E (2015). DAMP signaling is a key pathway inducing immune modulation after brain injury. J Neurosci.

[52] Liesz A, Zhou W, Mracsko E (2011). Inhibition of lymphocyte trafficking shields the brain against deleterious neuroinflammation after stroke. Brain.

[53] Liu F, Schafer DP, Mccullough LD (2009). TTC, fluoro-Jade B and NeuN staining confirm evolving phases of infarction induced by middle cerebral artery occlusion. J Neurosci Methods.

[54] Llovera G, Benakis C, Enzmann G (2017). The choroid plexus is a key cerebral invasion route for T cells after stroke. Acta Neuropathol.

[55] Ma S, Wang C, Mao X (2019). B cell dysfunction associated with aging and autoimmune diseases. Front Immunol.

[56] Manni M, Gupta S, Ricker E (2018). Regulation of age-associated B cells by IRF5 in systemic autoimmunity. Nat Immunol.

[57] Mcculloch L, Smith CJ, Mccoll BW (2017). Adrenergic-mediated loss of splenic marginal zone B cells contributes to infection susceptibility after stroke. Nat Commun.

[58] Meisel C, Meisel A (2011). Suppressing immunosuppression after stroke. N Engl J Med.

[59] Mittelbrunn M, Kroemer G (2021). Hallmarks of T cell aging. Nat Immunol.

[60] Monaco S, Nicholas R, Reynolds R et al. (2020) Intrathecal inflammation in progressive multiple sclerosis. Int J Mol Sci 21 10.3390/ijms21218217 PMC7663229 33153042

[61] Monson NL, Ortega SB, Ireland SJ (2014). Repetitive hypoxic preconditioning induces an immunosuppressed B cell phenotype during endogenous protection from stroke. J Neuroinflammation.

[62] Ning M, Furie KL, Koroshetz WJ (2006). Association between tPA therapy and raised early matrix metalloproteinase-9 in acute stroke. Neurology.

[63] Offner H, Subramanian S, Parker SM (2006). Experimental stroke induces massive, rapid activation of the peripheral immune system. J Cereb Blood Flow Metab.

[64] Ortega SB, Noorbhai I, Poinsatte K (2015). Stroke induces a rapid adaptive autoimmune response to novel neuronal antigens. Discov Med.

[65] Ortega SB, Torres VO, Latchney SE (2020). B cells migrate into remote brain areas and support neurogenesis and functional recovery after focal stroke in mice. Proc Natl Acad Sci USA.

[66] Pangrazzi L, Meryk A, Naismith E (2017). “Inflamm-aging” influences immune cell survival factors in human bone marrow. Eur J Immunol.

[67] Petereit HF, Pukrop R, Fazekas F (2003). Low interleukin-10 production is associated with higher disability and MRI lesion load in secondary progressive multiple sclerosis. J Neurol Sci.

[68] Pils MC, Pisano F, Fasnacht N (2010). Monocytes/macrophages and/or neutrophils are the target of IL-10 in the LPS endotoxemia model. Eur J Immunol.

[69] Pitzalis C, Jones GW, Bombardieri M (2014). Ectopic lymphoid-like structures in infection, cancer and autoimmunity. Nat Rev Immunol.

[70] Planas AM, Sole S, Justicia C (2001). Expression and activation of matrix metalloproteinase-2 and -9 in rat brain after transient focal cerebral ischemia. Neurobiol Dis.

[71] Prüss H, Iggena D, Baldinger T et al. (2012) Evidence of Intrathecal Immunoglobulin Synthesis in Stroke. Archives of Neurology 69 10.1001/archneurol.2011.3252 22371852

[72] Qin Y, Peng F, Ai L et al. (2021) Tumor-infiltrating B cells as a favorable prognostic biomarker in breast cancer: a systematic review and meta-analysis. Cancer Cell International 21 10.1186/s12935-021-02004-9 PMC8199375 34118931

[73] Rayasam A, Kijak JA, Kissel L et al. (2022) CXCL13 expressed on inflamed cerebral blood vessels recruit IL-21 producing TFH cells to damage neurons following stroke. Journal of Neuroinflammation 19 10.1186/s12974-022-02490-2 PMC9145182 35624463

[74] Ren X, Akiyoshi K, Dziennis S (2011). Regulatory B cells limit CNS inflammation and neurologic deficits in murine experimental stroke. J Neurosci J Society for Neurosci.

[75] Ritzel RM, Lai Y-J, Crapser JD (2018). Aging alters the immunological response to ischemic stroke. Acta Neuropathol.

[76] Rodriguez-Mogeda C, Rodriguez-Lorenzo S, Attia J et al. (2022) Breaching Brain Barriers: B Cell Migration in Multiple Sclerosis. Biomolecules 12 10.3390/biom12060800 PMC9221446 35740925

[77] Rost NS, Brodtmann A, Pase MP (2022). Post-Stroke Cognitive Impairment and Dementia. Circ Res.

[78] Saand AR, Yu F, Chen J (2019). Systemic inflammation in hemorrhagic strokes – A novel neurological sign and therapeutic target?. J Cereb Blood Flow Metab.

[79] Schafflick D, Wolbert J, Heming M (2021). Single-cell profiling of CNS border compartment leukocytes reveals that B cells and their progenitors reside in non-diseased meninges. Nat Neurosci.

[80] Schuhmann B, Dietrich A, Sel S (2005). A role for brain-derived neurotrophic factor in B cell development. J Neuroimmunol.

[81] Schuhmann MK, Langhauser F, Kraft P et al. (2017) B cells do not have a major pathophysiologic role in acute ischemic stroke in mice. Journal of Neuroinflammation 14 10.1186/s12974-017-0890-x PMC5457733 28576128

[82] Seifert HA, Vandenbark AA, Offner H (2018). Regulatory B cells in experimental stroke. Immunology.

[83] Selvaraj UM, Zuurbier KR, Whoolery CW (2018). Selective nonnuclear estrogen receptor activation decreases stroke severity and promotes functional recovery in female mice. Endocrinology.

[84] Sharma S, Yang B, Xi X (2011). IL-10 directly protects cortical neurons by activating PI-3 kinase and STAT-3 pathways. Brain Res.

[85] Simard JM, Kent TA, Chen M (2007). Brain oedema in focal ischaemia: molecular pathophysiology and theoretical implications. Lancet Neurol.

[86] Suzuki K, Maruya M, Kawamoto S (2010). Roles of B-1 and B-2 cells in innate and acquired IgA-mediated immunity. Immunol Rev.

[87] Tsao CW, Aday AW, Almarzooq ZI et al. (2022) Heart Disease and Stroke Statistics—2022 update: a report from the American Heart Association. Circulation 145 10.1161/CIR.0000000000001052 35078371

[88] Urra X, Cervera A, Villamor N (2009). Harms and benefits of lymphocyte subpopulations in patients with acute stroke. Neuroscience.

[89] Van Zwam M, Huizinga R, Melief MJ (2009). Brain antigens in functionally distinct antigen-presenting cell populations in cervical lymph nodes in MS and EAE. J Mol Med (Berl).

[90] Vega JA, Garcia-Suarez O, Hannestad J (2003). Neurotrophins and the immune system. J Anat.

[91] Wang S-S, Liu W, Ly D (2019). Tumor-infiltrating B cells: their role and application in anti-tumor immunity in lung cancer. Cell Mol Immunol.

[92] Wang Y, Chen D, Xu D (2021). Early developing B cells undergo negative selection by central nervous system-specific antigens in the meninges. Immunity.

[93] Wang Y, Liu J, Wang X (2017). Frequencies of circulating B- and T-lymphocytes as indicators for stroke outcomes. Neuropsychiatr Dis Treat.

[94] Weitbrecht L, Berchtold D, Zhang T (2021). CD4(+) T cells promote delayed B cell responses in the ischemic brain after experimental stroke. Brain Behav Immun.

[95] Xiao P, Chen Y, Jiang H (2008). In vivo genome-wide expression study on human circulating B cells suggests a novel ESR1 and MAPK3 network for postmenopausal osteoporosis. J Bone Miner Res.

[96] Yousefzadeh MJ, Flores RR, Zhu Y (2021). An aged immune system drives senescence and ageing of solid organs. Nature.

[97] Zbesko JC, Frye JB, Becktel DA (2021). IgA natural antibodies are produced following T-cell independent B-cell activation following stroke. Brain Behav Immun.

[98] Zhang D, Ren J, Luo Y (2021). T Cell Response in Ischemic Stroke: From Mechanisms to Translational Insights. Front Immunol.

[99] Zhou Y, Jiang Y, Peng Y (2017). The quantitative and functional changes of postoperative peripheral blood immune cell subsets relate to prognosis of patients with subarachnoid hemorrhage: a preliminary study. World Neurosurg.

[100] Zou Y-R, Davidson A (2018). Age-associated B cells acquire a new wrinkle. Nat Immunol.

